# Correction to “Dopamine‐Conjugated Extracellular Vesicles Induce Autophagy in Parkinson's Disease”

**DOI:** 10.1002/jev2.70082

**Published:** 2025-05-19

J. H. Sul, S. Shin, H. K. Kim, et al., “Dopamine‐Conjugated Extracellular Vesicles Induce Autophagy in Parkinson's Disease,” *Journal of Extracellular Vesicles* 13 (2024): e70018. https://doi.org/10.1002/jev2.70018

In the originally published article, Figure 5g contained an error. The DAPI and TH images of 6‐OHDA + Dopa‐EVs in Figure 5g were horizontally flipped. The corrected version of Figure 5g is provided below. This correction does not alter the description, interpretation or original conclusions of the article.


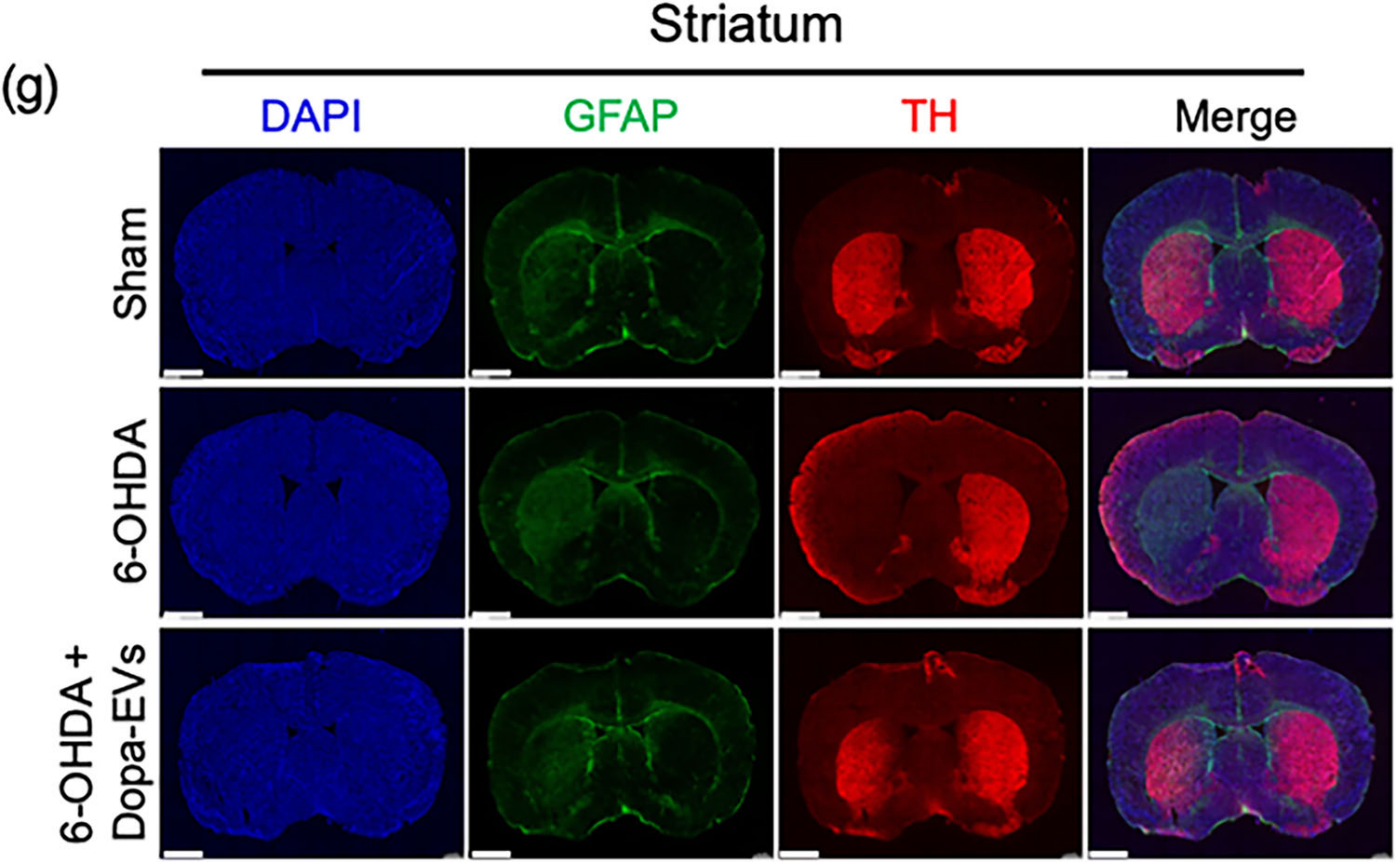


In Figure 5i, the y‐axis title was missing from the graph. The correct figure is as follows:


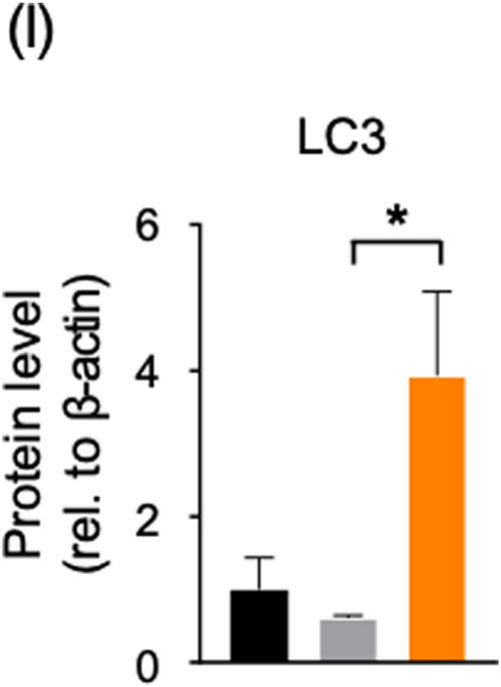


In Materials and Methods 2.11, the dosing schedule was described differently from Figure 5a, and an extra dash was added in the title. The correct title and text are as follows:

2.11 6‐OHDA‐induced PD mouse model

For injection of 6‐OHDA into the medial‐forebrain bundle, 8‐ to 10‐week‐old C57BL/6 male mice were deeply anaesthetized using a mixture of Zoletil (30 mg/kg) (Virbac) and Rompun (10 mg/kg) (Bayer) diluted at a 1:10 ratio with saline. They were then positioned in a stereotaxic mouse frame. 6‐OHDA (R&D Systems) was dissolved in 0.02% ascorbate (Sigma‐Aldrich)/saline solution at a concentration of 5 mg/mL and used within 3 h. The injection was administered at a rate of 0.2 µL/min into the medial forebrain bundle at the following coordinates (relative to the bregma): anterior–posterior (A/P) = −1.2 mm, mediolateral (M/L) = −1.2 mm, and dorsal‐ventral (D/V) = −4.85 mm (from the dura). After the injection, the syringe remained in place for an additional 5 min within the brain before being slowly withdrawn for a complete absorption of the solution. Control mice were injected with 0.02% ascorbic acid solution alone. To relieve mouse pain and improve survival rate after surgery, acetaminophen at a dose of 150 mg/kg was administered to mice twice a day for 3 days after injection of 6‐OHDA. After discrimination of PD‐induced mice using the pole‐test, EVs were administered intravenously (5 × 10 ^8^ particles/head) once daily for 7 consecutive days, starting 3 days after 6‐OHDA injection.

In Materials and Method 2.15, the antibody information listed in the manuscript was incorrect. The updated text is as follows:

… Membranes were blocked in 5% non‐fat milk for 1 h at room temperature, and incubated overnight at 4°C with antibodies raised against Calnexin (AB2301, Sigma‐Aldrich), TSG101 (sc‐7964, Santa Cruz Biotechnology), CD9 (sc‐13118, Santa Cruz Biotechnology), β‐actin (A2228, AC‐74, Sigma‐Aldrich), CD63 (MX‐49.129.5, Santa Cruz Biotechnology), tyrosine hydroxylase (2792, Cell Signaling Technology), Parkin (ab77924, clone Prk8, abcam), LC3B (NB100‐2220, Novus Biologicals), NRF2 phospho (Ser40) (2073‐1, Epitomics), α‐synuclein (610787, clone 42/α‐Synuclein, BD biosciences), Beclin‐1 (A11761, ABclonal), Tubulin β3 (TUBB3) (MMS‐435P, clone TUJ1, Biolegend), Akt (9272, Cell Signaling Technology), Phospho‐Akt (Ser473) (9271, Cell Signaling Technology), Phospho‐GSK‐3β (Ser9) (5558, D85E12, Cell Signaling Technology), GSK‐3α/β (sc‐7291, 0011‐A, Santa Cruz Biotechnology), Phospho‐p44/42 MAPK (Erk1/2) (Thr202/Tyr204) (4370, clone D13.14.4E, Cell Signaling Technology) and p44/42 MAPK (Erk1/2) (4696, clone L34F12, Cell Signaling Technology). …

In Results 3.5, the sentence was incorrect. The terminology referring to the substances or components inside vesicles was misspelled. The correct text is as follows:

… Intriguingly, disrupting the membrane integrity of Dopa‐EVs abolished these anti‐Parkinson's effects (Figure 7g), indicating that the therapeutic efficacy of Dopa‐EVs is mediated by their intravesicular contents. …

We apologize for these errors.

